# Trends in Vascular Access Among Patients Initiating Hemodialysis in the US

**DOI:** 10.1001/jamanetworkopen.2023.26458

**Published:** 2023-08-01

**Authors:** Michael Allon, Yi Zhang, Mae Thamer, Deidra C. Crews, Timmy Lee

**Affiliations:** 1 Division of Nephrology, Department of Medicine, University of Alabama at Birmingham; 2 Medical Technology and Practice Patterns Institute, Bethesda, Maryland; 3 Division of Nephrology, Department of Medicine, Johns Hopkins University School of Medicine, Baltimore, Maryland; 4 Division of Nephrology, Veterans Affairs Medical Center, Birmingham, Alabama

## Abstract

This cohort study uses the US Renal Data System database to analyze trends in vascular access among more than 600 000 patients who initiated hemodialysis from 2015 to 2020.

## Introduction

The 2006 clinical practice guidelines for vascular access recommend placing arteriovenous fistulas (AVFs) in patients with advanced chronic kidney disease prior to need for maintenance hemodialysis, to decrease central venous catheter (CVC) dependence.^1^

## Methods

In this cohort study, we analyzed the US Renal Data System database in May 2023 and identified 639 883 patients who initiated hemodialysis from January 1, 2015, to December 31, 2020. We assessed which vascular access type (AVF, arteriovenous graft [AVG], or CVC) was used at dialysis initiation. Patients who initiated dialysis with an AVF or a CVC and a maturing AVF were considered to have undergone predialysis AVF placement. Changes in initial vascular access use over the 6-year period were adjusted for baseline patient characteristics. The Cochran-Armitage trend test was used to assess changes in proportions of vascular access availability in each year and by range of predialysis nephrology care. All *P* values were from 2-sided tests, and results were deemed statistically significant at *P* < .05. Our report follows the STROBE reporting guideline. The University of Alabama at Birmingham institutional review board waived a review and patient consent because we used deidentified information.

## Results

During the 6 years of the study, of 639 883 patients (58% men and 42% women; mean [SD] age, 63 [15] years), approximately 100 000 to 110 000 patients initiated hemodialysis annually. There were minimal changes during this period in patient demographic characteristics, comorbidities, functional status, or insurance type; however, predialysis nephrology care increased from 76% (81 308 of 107 033) to 82% (84 344 of 102 662).

**Figure 1.**
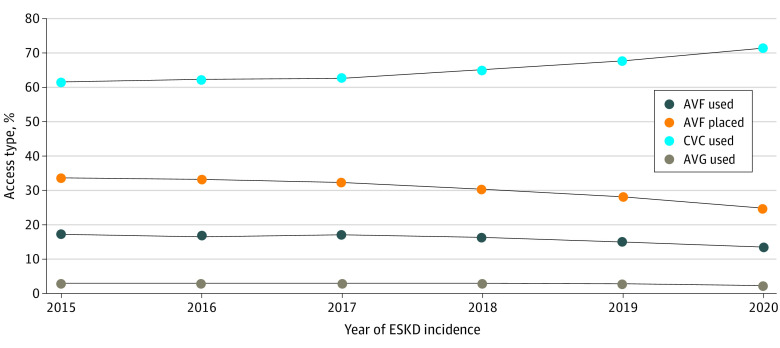
Temporal Trends From 2015 to 2020 in the Proportion of Patients With Arteriovenous Fistula (AVF) Use, Arteriovenous Graft (AVG) Use, Central Venous Catheter (CVC) Use at Dialysis Initiation, and Predialysis AVF Placement *CVC use* refers to patients with a CVC only and no AVF or AVG at the time of dialysis initiation. *P* < .001 for trend in all access types except AVG. ESKD indicates end-stage kidney disease.

AVF use at dialysis initiation decreased from 17% (18 600 of 107 033) in 2015 to 14% (14 060 of 102 662) in 2020 (18% relative decline) (Figure 1). This decrease occurred across multiple patient subgroups, including those defined by age, sex, race and ethnicity, comorbid disease burden, functional status, and health insurance coverage. Predialysis AVF placement decreased from 34% (36 143 of 107 033) in 2015 to 25% (25 413 of 102 662) in 2020 (26% relative decrease). The proportion of patients with predialysis AVF placement who subsequently used their AVF at dialysis initiation increased from 51% (18 600 of 36 143) in 2015 to 55% (14 060 of 25 413) in 2020, indicating a higher AVF maturation rate. Concurrently, CVC use at dialysis initiation increased from 61% (65 802 of 107 033) in 2015 to 71% (73 362 of 102 662) in 2020 (16% relative increase), and AVG use remained constant at 3% (3307 of 107 033 patients in 2015 and 2689 of 102 662 patients in 2020). Trends in AVF and CVC use at dialysis initiation were accelerated with onset of the COVID-19 pandemic in 2020. Compared with 2015, the adjusted odds ratio was 0.71 (95% CI, 0.69-0.73) for initiation of hemodialysis with an AVF and 1.72 (95% CI, 1.69-1.75) for initiation with a CVC in 2020.

**Figure 2.**
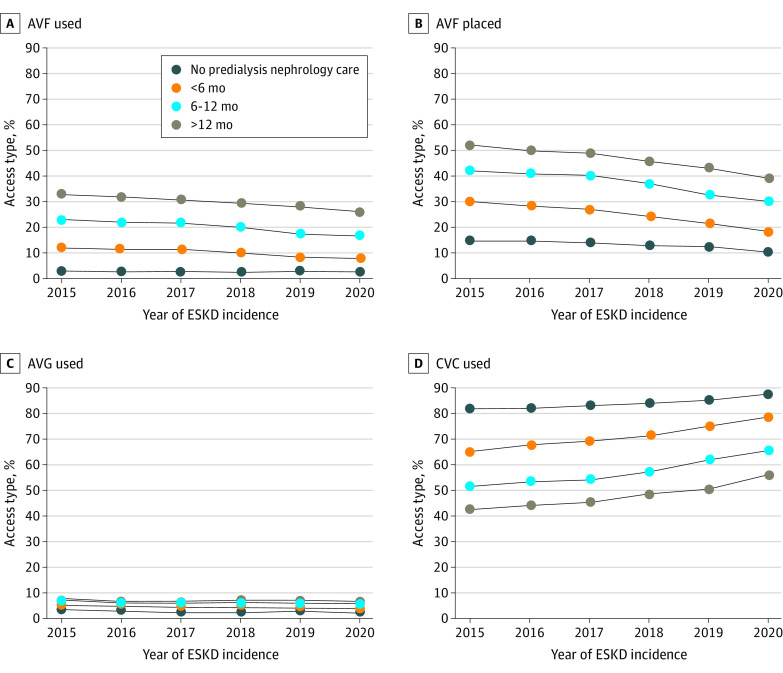
Association of Duration of Predialysis Nephrology Care With the Likelihood of Using an Arteriovenous Fistula (AVF), Arteriovenous Graft (AVG), or Central Venous Catheter (CVC) at Initiation of Hemodialysis and the Likelihood of Undergoing Predialysis AVF Placement *CVC use* refers to patients with a CVC only and no AVF or AVG at the time of dialysis initiation. Longer duration of predialysis nephrology care was associated with a higher likelihood of starting hemodialysis with an AVF and a lower likelihood of initiating hemodialysis with a CVC, but it was not associated with AVG use. However, regardless of duration of predialysis nephrology care, AVF use decreased and CVC use increased between 2015 and 2020. ESKD indicates end-stage kidney disease.

With a logistic regression model including demographic charactristics, comorbidities, functional status, health insurance, and duration of predialysis nephrology care, the most important factor associated with AVF placement and use was duration of predialysis nephrology care; regardless of duration, AVF use decreased and CVC use increased at dialysis initiation between 2015 and 2020. However, the longer the duration of nephrology care, the higher the likelihood of using an AVF and the lower the likelihood of using a CVC at initiation of dialysis (Figure 2).

## Discussion

AVF use at dialysis initiation decreased from 2015 to 2020. This trend was not associated with changes in patient demographic or clinical characteristics, a concurrent increase in AVG placement, a lower likelihood of predialysis nephrology care, or a lower rate of AVF maturation. It also was not associated with a heightened reluctance to place AVFs in patients with kidney failure because, during this same period, the proportion of patients who have been undergoing hemodialysis using an AVF or CVC remained constant at 55% and 18%, respectively.^2^

We hypothesize 3 explanations for the decrease in AVF use among patients initiating hemodialysis: less emphasis on predialysis vascular access management compared with its management among patients who have been undergoing dialysis, a greater focus on shared decision-making including whether to pursue dialysis (rather than conservative medical management)^3^ and whether to pursue peritoneal dialysis (rather than hemodialysis)^4^ before referral for vascular access surgery, and more selective placement of AVFs in patients for whom an AVF is likely to succeed.^5^ The decrease in AVF use may be the inadvertent outcome of the more nuanced, patient-centered 2019 vascular access guidelines, which emphasize selection of the access that is most suitable for each patient.^6^ Our analysis is limited by use of administrative data.

## Notes

Supplement.
Data Sharing Statement
