# Supplementary material for: Trends in Vascular Access Among Patients Initiating Hemodialysis in the US
Source: JAMA Netw Open. 2023 Aug 1;6(8):e2326458. doi: 10.1001/jamanetworkopen.2023.26458 (PMC10394578; doi:10.1001/jamanetworkopen.2023.26458)
Supplement: Supplement. — Data Sharing Statement [file jamanetwopen-e2326458-s001.pdf]

Data Sharing Statement

Allon. Trends in Vascular Access Among Patients Initiating Hemodialysis in the US. *JAMA Netw Open*. Published August 01, 2023. doi:10.1001/jamanetworkopen.2023.26458

Data
Data available: No

Additional Information
Explanation for why data not available: The USRDS databases is available to any investigator following application and approval.
